# The effects of psychedelics on attention-deficit and hyperactivity disorder – a systematic review

**DOI:** 10.1017/neu.2026.10088

**Published:** 2026-06-10

**Authors:** Helerin Raikkerus, Andrea Bujor, Mark Kennedy, Jari Tiihonen

**Affiliations:** 1 Helsinki University Hospital Physiatry, Finland; 2 King’s College London Institute of Psychiatry Psychology & Neuroscience, UK; 3 Department of Clinical Neuroscience, Karolinska Institute, Sweden; 4 Centre for Psychiatry Research, Stockholm Health Care Services, Sweden; 5 Department of Forensic Psychiatry, Niuvanniemi Hospital, University of Eastern Finland, Finland; 6 Neuroscience Center, University of Helsinki, Finland

**Keywords:** attention-deficit disorder with hyperactivity, ADHD, hallucinogens, ketamine, LSD

## Abstract

**Objectives:**

Attention-deficit and hyperactivity disorder (ADHD) is a prevalent neuropsychiatric disorder. Recently, psychedelics have become of interest regarding developing treatment options for ADHD. The aim of this systematic review is to find all studies from the APA PsychInfo and MEDLINE databases, where psychedelics have been used for ADHD and assess whether further clinical studies are warranted.

**Methods:**

APA PsychInfo and MEDLINE were searched on the 20th of August 2025 for studies discussing ADHD and lysergic acid diethylamide (LSD) or psilocybin or dimethyltryptamine (DMT) or mescaline or phencyclidine or 3,4-methylenedioxymethamphetamine (MDMA) or ketamine. Primary research articles in English where the effects of the psychedelics mentioned on ADHD in humans were included.

**Results:**

*N* = 1023 results were identified. Six studies were included – one randomised controlled trial (RCT) finding no statistically important difference compared to placebo, three cross-sectional studies where respondents reported positive effect of psychedelics and one where the statistically important improvement was measured by the Child Bipolar Questionnaire. In addition, one case study, where both, depressive symptoms and functioning improved with ketamine.

**Conclusions:**

There is not sufficient evidence to give recommendations on psychedelic use for ADHD. In addition, it is not known whether patients, whose depressive symptoms have responded positively to ketamine, have also had ADHD. Also, no research was found on how psychedelics affect patient subgroups with different etiopathology causing their symptoms. Although only six studies filled the inclusion criteria, they bring out valuable implications for further research.

## Graphical abstract

**Figure:**
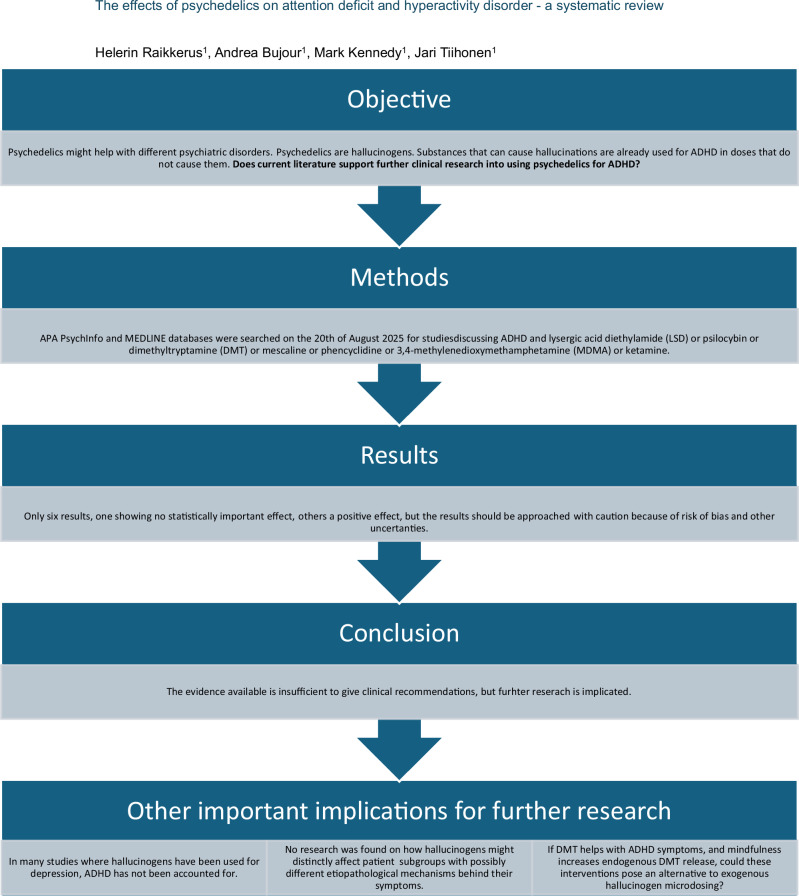

Significant outcomes

- There is not sufficient evidence to give recommendations on psychedelic use for ADHD. The evidence is inconclusive and scarce.
- In many studies where psychedelics have been used for depression, ADHD has not been accounted for. Therefor it is not known whether patients, whose depressive symptoms have responded positively to ketamine, also have suffered from ADHD.
- No research was found on how psychedelics might distinctly affect patient subgroups with possibly different etiopathological mechanisms behind their symptoms.

Limitations

- The search was limited only to the APA PsychInfo and MEDLINE databases.
- From *N* = 1023 screened articles only six publications filled the inclusion criteria.
- Three out of six included studies analysed self-reported, possibly biased, data.

Attention-deficit and hyperactivity disorder (ADHD) is thought to be a prevalent developmental neuropsychiatric disorder that can inflict significant distress (Drechsler *et al*., 2020). There are some treatment options available, for adults the main positive effects have been shown for stimulants and atomoxetine (Ostinelli *et al*., 2025). Nevertheless, not all patients benefit from the approved medications, or the drugs are contraindicated for possible side effects (Ostinelli *et al*., 2025; Reeves & Tickle, 2025). Because of this, new types of treatment are needed. If these new types of treatment would help people who cannot use conventional treatment or would be safer and/or more specific than the conventional ones, society could potentially benefit even more.

One type of drugs of interest regarding developing new treatment options for ADHD are psychedelics. The possibly beneficial effect of some psychedelics has been shown for some other psychiatric conditions such as depression, post-traumatic stress disorder (PTSD) and alcohol use disorder (Luquiens *et al*., 2025). Psychedelics can be divided into classical and non-classical psychedelics. The term classical psychedelics is widely used for substances that primarily exert their psychedelic effect through agonism or partial agonism at the brain serotonin 5-hydroxytryptamine (5-HT) 2A receptors (De Gregorio *et al*., 2018). Some examples are lysergic acid diethylamide (LSD), dimethyltryptamine (DMT), psilocybin and mescaline (De Gregorio *et al*., 2018). In a broader sense, classical psychedelics belong to a wider group of hallucinogens or psychotomimetic drugs that can be understood as all substances able to cause a psychosis-like state (Nichols, 2016). Drugs, that fit the definition are, for example, 3,4-methylenedioxy-methamphetamine (MDMA) and ketamine (Nichols, 2016). In addition, this definition would also include stimulant drugs, that are already widely used in the treatment of ADHD at a dosage that does not generally cause psychosis (Ching *et al*, 2019). This makes using conventional ADHD medications comparable to taking regular low dosages of psychedelic drugs or microdosing. This review focuses on classical psychedelics, and other widely used hallucinogens that are often discussed alongside them, collectively referring to them as psychedelics (Nichols, 2016).

Different mechanisms behind how psychedelics might help with ADHD symptoms have been proposed in the literature. Most psychedelics, in addition to drugs routinely used in the treatment of ADHD, can have a stimulatory effect on the central nervous system (Ninan *et al*., 2003; Felsch & Kuypers, 2022). Classical psychedelics are thought to exert their main psychedelic effects through the activation 5HT2A receptors, but can, depending on the drug, also influence other receptors, for example other serotonin- or dopaminergic receptors, trace amine-associated receptors (De Gregorio *et al*., 2018). This activation of 5HT2A receptors can have a stimulatory effect through increasing cortical glutamate release (De Gregorio *et al*., 2018). Ketamine is widely hypothesised to work mainly through inhibitory effects on N-methyl-d-aspartate receptors (NMDARs) on interneurons also leading to increased cortical glutamate release (Zorumski *et al*., 2016). MDMA has both amphetamine-like effects and additional effects on the serotoninergic system (it resembles mescaline chemically) (Kalant, 2001). For example, Hutten *et al*. (2024) have comprehensively reviewed neural responses to low doses of LSD in healthy participants. They found that LSD had a stimulatory effect and that the effect was best in people with lower arousal at baseline (Hutten *et al*., 2024). Also, users of psychedelics have claimed that they can help with ADHD symptoms and positive effects have been found in not placebo-controlled studies (Haijen *et al*., 2022). Nevertheless, information from preclinical studies also points to other possible neurobiological mechanisms behind possible positive effects regarding ADHD treatment. One of these is increased neuroplasticity. Interestingly, in recent literature neuroplasticity has also been implicated as one possible mechanism behind how stimulants already used in ADHD treatment might work (Contreras *et al*., 2022; Mastinu *et al*., 2023). In addition, some effects could be mediated by epigenetic effects (Song *et al*., 2025). Increased connectivity in functional brain networks, especially between the prefrontal and parietal cortices and salience network, and increased barriers for rapid global shifts have also been implicated as possible mechanisms behind the psychological effects of psychedelics (Gattuso *et al*., 2023; Shukuroglou *et al*., 2023; Blackburne *et al*, 2025; Sabbah *et al.*, 2026). Even though there are ample of possible ways how psychedelics could be of benefit, it has also been argued that psychedelics do not help with ADHD. For example, Mueller *et al*. (2025) recently published the results of a randomised controlled trial (RCT) where LSD microdosing was used for ADHD symptoms, and they did not find a statistically significant difference between ADHD symptom improvement and LSD microdosing compared to placebo. In addition, positive results reported by not placebo-controlled studies could be explained by placebo effect and psychedelics usage among drug users with or without ADHD seems to be similar (Tschudi *et al*., 2023). In addition to the possibility of that these drugs might not be of benefit; it is important to remember that there could also be safety risks involved. The safety of different psychedelics has been widely investigated, and preliminary results do not preclude the potential use of this drug, if the risks are consciously accounted for (Perry *et al*, 2007; Smith *et al*., 2022; Bender & Hellerstein, 2022).

Studying the effects of psychedelics on ADHD symptoms is difficult Firstly, there might be different neurobiological mechanisms behind ADHD symptoms, and these mechanisms are not yet entirely understood (Sonuga-Barke *et al*., 2003; Kennedy *et al*., 2016; Chen *et al*., 2025). Secondly, different psychedelics have different psychopharmacological profiles. Also, they can be administered differently, possibly affecting the outcomes. Sometimes the quality of the drugs used, can be of medical grade, other times it is not possible to determine the dosages and substances used (for example in case of using ‘magic mushrooms’ or ayahuasca tea) (Mastineu *et al*., 2023). In case of differences in dosages, as in the case of psilocybin, small doses might increase neurogenesis, but higher doses might be detrimental (de Vos *et al*., 2021). Similar dose sensitivity of effects has been shown for example for LSD, mescaline (Murray *et al*, 2022; Mastinu *et al*., 2023). In addition to uncertainties related to the continents of uncontrolled products, the route of administration can be of pharmacological importance. In addition, additives can have important effects on the outcomes – for example if DMT containing leaves from *Psychotria viridis* are consumed with monoamine oxidase inhibitors from *Banisteriopsis caapi*, the hallucinogenic effects can be potentiated because more DMT reaches the central nervous system (Barker, 2022). These uncertainties in psychedelic research complicate the appraisal of the clinical significance of studies, where the active substances used are unknown. Still, even though a complicated area of research – could some hallucinogens, in addition to known ADHD medications, be also used in the treatment of ADHD? If so, are there be different subtypes of ADHD that would potentially benefit from psychedelics treatment? It is difficult to find literature on this subject. A scoping or systematic review on the subject could give more answers.

No scoping or systematic reviews have been published on the effects of different psychedelcis in treating ADHD although this is a subject of increasing interest in the scientific community. The aim of this systematic review is to find all studies from the APA Psychinfo and MEDLINE databases, where people suffering from ADHD have used psychedelics for the symptoms caused by the disorder to help to assess whether further clinical studies are warranted.

## Methods

The PRISMA guidelines for systematic reviews were followed throughout the process (Page *et al*., 2021). This review was pre-registered on Prospero (Raikkerus *et al*., 2025). The search criteria were chosen collectively by the collaborating team to reflect the most used psychedelics and hallucinogens in clinical psychiatry or research. Other, broader terms, such as psychedelics, hallucinogens,but also for example ayahuasca (contains DMT) or ‘magic mushrooms’ (can contain psilocybin) were omitted, because in psychopharmacological studies the active ingredients of medication are usually mentioned and more exactly determined. Overly inclusive terminology may amplify the uncertainty of findings as the composition might be inconsistent (Callaway, 2005; Kuchar *et al*., 2025). It was decided to include MDMA and ketamine even though they are not considered classical psychedelics, they can be considered psychedelics under a broader definition and are frequently discussed alongside classical psychedelics (Nichols, 2016). APA PsychInfo and MEDLINE were searched on the 20th of August 2025. The following string was used (incorporating Medical Subject Headings (MeSH) in MEDLINE alongside free-text keywords):

((adhd or attention or hyperactivity) and (lysergic acid diethylamide or lsd or psilocybin or Dimethyltryptamine or DMT or mescaline or phencyclidine or 3,4-methylenedioxy-methamphetamine or MDMA or ketamine))

**Figure 1.**
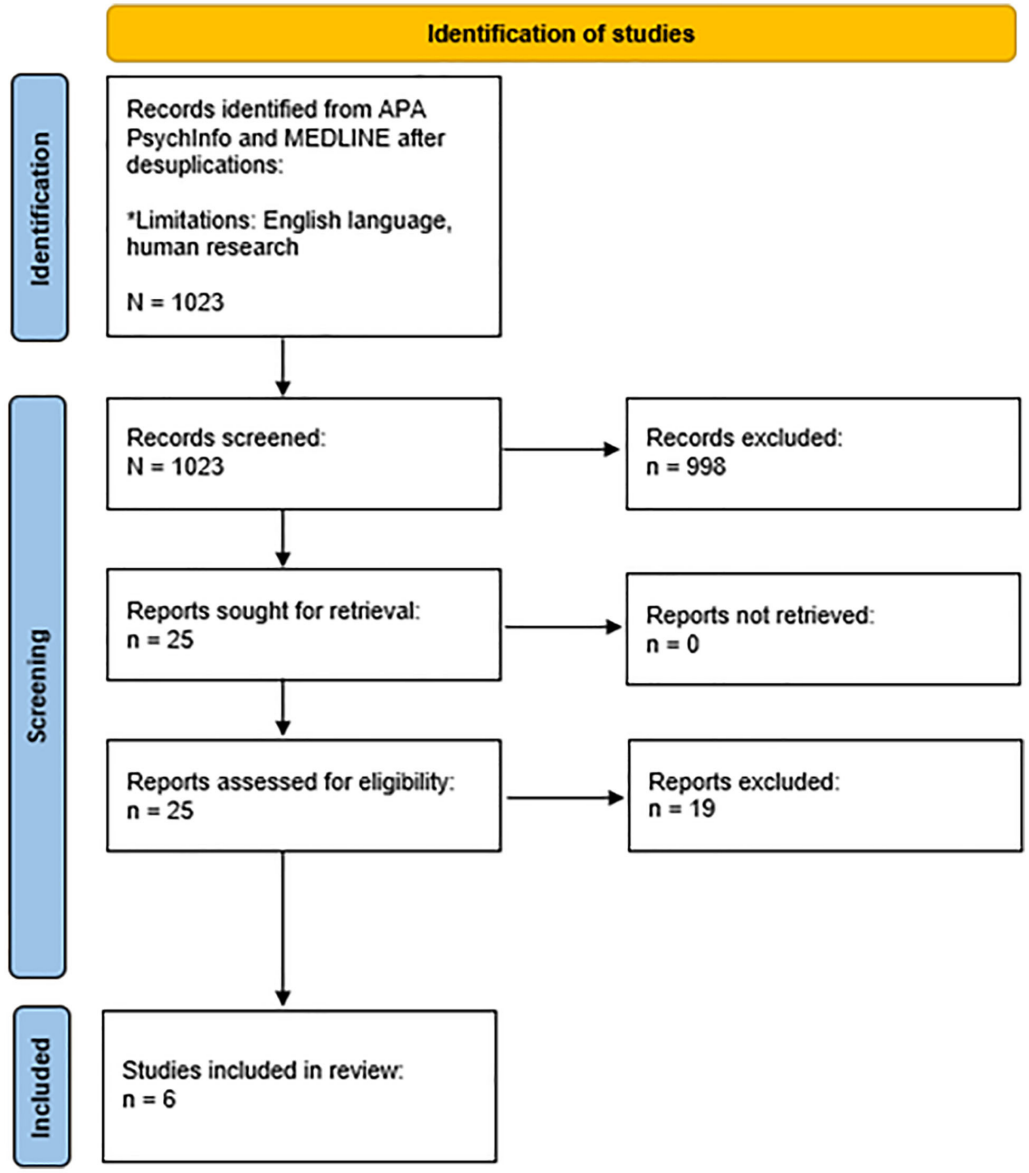
Prisma Flowchart. *Note:* Adapted from Page *et al.* (2021).

Primary research articles where the effects of the psychedelics mentioned on ADHD were assessed. Because research on the area is scarce, self-report data was also allowed acknowledging the risk of bias separately. Only research published in English was included. No timeframe restrictions were made. Only primary studies involving humans were included. Non-peer-reviewed literature, conference abstracts, editorials were excluded. The papers were excluded if only symptoms of ADHD were looked at with no reference to clinical ADHD or it was not possible to deduce from the text how psychedelics were found to influence the symptoms. Two authors screened all the search results showing a moderate level of agreement (Cohen’s *κ* = 0.44). The academic background of the screeners was different, even though both have a Master of Science degree in psychology, one had a wider background in clinical psychiatry as a physician. Then again, all the studies, that the two raters had not both included independently, were discussed with the other authors. Disagreements were resolved through discussion before a final decision was made. AI-assisted screening tool Rayyan was used to facilitate inter-reviewer collaboration during the screening process (Ouzzani *et al*., 2016). Figure 1 summarises the screening and eligibility assessment using the PRISMA flowchart (Page *et al*., 2021).

Data was extracted and validated by one member of the collaborating team. Studies were grouped by study design. Quality of the studies and risk of bias were critically assessed using the appropriate Critical Appraisal Skills Programme (2024) checklist according to study type and the results summarised using narrative synthesis. This study summarises already published data available on the Internet and does not require a separate Ethics Assessment according to the Finnish National Board on Research Integrity (2019) guidelines.

## Results

Out of *N* = 1,023 studies only six fulfilled the inclusion criteria. Only one was a RCT (Mueller *et al*., 2025). Papolos *et al*. (2013) did a retrospective chart analysis. Three others used self-report data from the internet or surveys (Hutten *et al*., 2019; Lea *et al*., 2020; Natter & Michel, 2020). In addition, one case study was identified (Dwyer *et al*., 2017). Many studies picked up by the search were excluded, because the patients were only assessed for changes in depressive symptoms, and the participants were not assessed for possible ADHD as a comorbidity. Even though ADHD is often found in people with treatment resistant depression, the collaborating team decided to exclude these studies, because treatment resistant depression can be associated with many different psychiatric disorders and analysing all is beyond the scope of this review (Sternat *et al*., 2018; Fabbri *et al*., 2021). Regarding studies looking at the effects of psychedelics on affective symptoms, it was decided to include only the studies where ADHD was assessed. The results are shown in Table 1.

**Table 1.** Included studies and their characteristics

Study	Population	Intervention	Control	Outcomes
**Mueller *et al*., 2025** (RCT)	*N* = 53 adults aged 18–65 years with moderate to severe ADHD and without other major psychiatric or somatic disorders	Lysergic acid diethylamide (LSD) microdosing, dosage 20 µg	Placebo group	Improvement of symptoms in both groups, no statistically important difference between groups.
**Lea *et al*., 2020** (Cross-sectional study)	*N* = 1102 respondents who reported having microdosed psychedelics, out of them 19.5% of respondents reported also being diagnosed with ADHD	LSD microdosing, median microdose 11 µg (interquartile range 10–20 µg). *The dosages were inexact and subjectively assessed by participants	–	10.9% of respondents reported ADHD treatment as their primary or secondary motivating factor for microdosing.
**Hutten *et al*., 2019** (Cross-sectional study)	*N* = 1122 adults who reported having microdosed psychedelics, out of them a subgroup of *n* = 410 patients reported also being diagnosed with at least one mental disorder	Self-reported use of psychedelics with self- assessed and self-reported dosage	Same participants with conventional therapy, microdosing compared to macrodosing	Self-reported microdosing was assessed more effective than conventional treatment regarding ADHD (OR = 11.66, 95% CI [3.46, 39.34], *p* < 0.01), but no statistically significant difference was found between the effects of microdosing and microdosing.
**Papolos *et al*., 2013** (Cross-sectional study)	12 youth, 10 males 2 females aged 6–19 years, with treatment-refractory bipolar disease with fear of harm phenotype	Intranasal ketamine in a clinical setting, dosage ranged from 30 to 120 mg every d 3–7 days according to clinical response and indication.	Same patients before treatment	Ketamine was associated with improvements in different symptoms, incl. attention/executive functions assessed by the Child Bipolar Questionnaire (CBQ) (<0.0001).
**Natter & Michel, 2020** (Cross-sectional study)	136 Reddit.com users	Self-reported use of memantine with self- assessed and self-reported dosage	–	11 respondents (8.8%) reported using memantine for ADHD.
**Dwyer *et al*., 2017** (case study)	16-year-old male, with major depressive disorder, ADHD and Crohn’s disease	Week 1: ketamine 0.5 mg/kg/40 min 3 times a weekWeeks 2–5: same dose, once a weekRecovery phase (outpatient): from once every 3–6 weeks and increasing intervals	–	Depressive symptoms and functioning improved. 61% MADRS score reduction after the first infusion. The patient recovered from depression and was discharged, after week 5 it was possible give infusions at an outpatient clinic.

In the study conducted by Mueller *et al*. (2025) the study questions were clearly formulated and methodology clearly presented. Participants were randomised using computer-generated randomisation with balanced blocks and the allocation was blinded from the investigators. Participants in the intervention group almost unanimously guessed the allocation (96%) compared to the control group (33%) indicating that participant blinding was not efficient. All the participants received the same level of care. Appropriate statistical methods were used and the results clearly presented. In both study groups ADHD symptoms declined and no statistically important difference between the intervention and the control groups was found. The power of the study was calculated based on *n* = 26 participants per group and found to be 80%. Considering that only *n* = 53 people out of *N* = 503 contacted by phone or email entered the study, there could still be a lot of information missing and the results biased. The study methodology was of good quality, but the results must be treated with caution.

The results of cross-sectional studies mainly analysing self-reported or results not clearly attributable to ADHD symptoms and should be treated with caution. Three out of four studies analysed self-reported data. All three studies were well designed, appropriate analytic methods were used, ethical aspects were considered and the results were clearly reported (Hutten *et al*., 2019; Lea *et al*., 2020; Natter & Michel, 2020). Even though the psychedelic considered by Natter & Michel, 2020, was memantine, that was not included in the original search string, it was included because memantine can be considered a psychedelic using the broader definition (Nichlas, 2016). Nevertheless, it is important to note that there might be a selection bias towards participants with positive experiences and only self-reported data was used for analysis. Also, not knowing the quality or exact dosages of the drugs used makes the results less reliable. Still, the results show that some people use psychedelics for ADHD symptoms. The study by Papolos *et al*. (2013) differed from other cross-sectional studies. They analysed data from 12 youth with treatment-refractory bipolar disorder with a paediatric-onset fear of harm (FOH) phenotype (Papolos *et al*., 2013). The power of the study is low, but it can be interpreted as a pilot study. There is a risk for selection bias (from a private praxis), the relationship between the researchers and the patients was not discussed and no blinding was used. Ethics was considered adequately. The Child Bipolar Questionnaire (CBQ) results improved, but the results are inexact as the measure use has different dimensions (inc. depressive and ADHD symptoms). Also, it is well known (Comparelli *et al*., 2022), that differential diagnostics between bipolar disorder and ADHD is difficult in children. ADHD symptoms might have improved, but it is difficult to assess, to which extent (Papolos *et al*., 2013). The value of the study by Papolos *et al*. (2013) is in implications for further research on further elucidating the neurobiological mechanisms on the effects of ketamine on different conditions.

One case study was identified. Dwyer *et al.* (2017) looked at neurocognitive changes in a patient with major depressive disorder, but also ADHD, and had received ketamine treatment. Some symptoms of ADHD and major depressive disorder overlap, and it is difficult to differentiate whether treatment also helped with ADHD symptoms. Then again, it is said in the article that neurocognitive assessments remained stable in the recovery phase (information not given for acute phase) (Dwyer *et al*., 2017). The limitations of the article were, that it looked at only one patient, making the results less generlisable, the relationship between the researcher and the participant had not been adequately considered and even though it is mentioned that an informed consent was given for the treatment, it is not mentioned whether consent for publication was sought. Still, the information is valuable as the treatment helped with depressive symptoms and indicates further research on differentiating the effects of psychedelics on depression and ADHD.

## Discussion

ADHD is thought to be a prevalent developmental neuropsychiatric disorder that can inflict significant distress and current treatment options are limited (Drechsler *et al*., 2020; Ostinelli *et al*., 2025; Reeves & Tickle, 2025). The aim of this systematic review was to find all studies from the APA Psychinfo and MEDLINE databases, where people suffering from ADHD have used psychedelics (in a wider definition) for the symptoms caused by the disorder to assess whether further clinical studies are warranted.

The evidence on the subject was scarce. Only one RCT was found and these results should be approached with caution because of possibly insufficient dosage, blinding, missing information and risk of bias. Most of the other studies were cross-sectional and were based on self-reported data exposed to the risk of subjective reporting and should also be approached with caution. No studies that would have looked at the effects of psychedelics separately in different subpopulations with possibly different etiopathological mechanisms behind their symptoms were not found. One case study was identified, but the results are difficult to interpret in terms of ADHD symptom change and not easily generalisable.

A limitation of this study was that only two databases were searched, even though MEDLINE and PsychInfo are two of the main databases for medical and psychological research, accordingly. Also, only a narrow, although commonly used, set of search words were used. Wider terms as psychedelics, or hallucinogens, or magic mushrooms and Ayahuasca were omitted for attaining more precise results. Results indexed in only other databases or that might have been caught with a wider search were not included in this review and it might have missed some publications that could have fulfilled the inclusion criteria. For instance, the study conducted by Haijen *et al.* (2022) was not retrieved through the search. Even though it was an intervention study, it does not change the conclusions of this systematic review. In addition, as only six studies were included, there were resources for throughout appraisal.

There is not sufficient evidence to give recommendations on hallucinogen use for ADHD. On the other side, there is ample evidence from preclinical studies that psychedelics can have a positive effect on ADHD symptoms and many suffering from ADHD symptoms have found them effective. Then again, many of these studies relied on possibly biased, subjective, self-report data (Hutten *et al.*, 2019; Lea *et al*., 2020; Natter & Michel, 2020). Although only a few studies could be included in this systematic review, the results are important because of clear indications for future research.

No research was found on how psychedelics can influence patients with different subtypes of ADHD. For example, lately there have been indications that childhood adverse experiences (ACEs) can also cause ADHD (Raikkerus, 2025). This population might especially benefit from new treatments because patients with dual diagnosis of PTSD and ADHD are at a heightened risk for psychosis and stimulants may contribute to psychosis risk (Gelner *et al*., 2023; Oliva *et al*., 2025). In addition, some psychedelics have been found effective in treating PTSD (De Gregorio *et al*., 2021). Coming back to the study by Papolos *et al*. (2013) who looked at how ketamine helped children with bipolar disorder with FOH phenotype. Depressive symptoms improved, ADHD symptoms improved, unfortunately ACEs were not assessed (Papolos *et al*., 2013). In addition to diagnostic difficulties between ADHD and bipolar disorder in childhood (Wozniak *et al*., 2025), it has been shown that childhood adversity can affect the clinical characteristics of bipolar disorder presentation (Etain *et al*., 2013). This, together with previous results, implicate the need for further research on psychedelic treatment for ADHD, especially in possible different etiopathological subgroups.

A major strength of this study was looking at ketamine and MDMA alongside the classical psychedelics and an interdisciplinary research team. Because of this another research gap was identified. In many studies, that were excluded from this systematic review, some psychedelics had a positive effect on depression. Even though it is known that many suffering from treatment resistant depression, might have comorbid ADHD (Sternat *et al*., 2018), it was usually not assessed in the search results. This means that, even though it has been repeatedly shown that, for example, ketamine can have positive effects on treatment resistant depression, it is largely not known, how many people in the samples studied also suffered from ADHD and how ketamine treatment affected the ADHD symptoms. A search in MEDLINE alone, using depression and ketamine as keywords, gave 5,987 results on the 12th of October 2025.

There have been some concerns about the safety of using psychedelics as treatment. In addition to psychopharmacological effects, it has been proposed that one mechanism behind helping with depressive symptoms might be the loss of fear of death (Letheby, 2024). Which raises difficult ethical questions from patient consent and study design to long term follow up needs. Then again, for example ketamine, is already widely used clinically in the treatment of depression, especially if other options have failed or cannot be used. Particularly in these cases the need for other treatment options arises. Mostly, the evidence has shown that psychedelics used in treatment are well tolerated, with a low risk of serious injury (Romeo *et al.*, 2024). Still, new treatment options should be approached with caution always assessing the risk and benefit ratio in every individual patient or study participant.

One interesting possible direction for future research would be the studying the effects of endogenous DMT. It is known that mindfulness-type interventions can have a positive effect on attention (Gonzales *et al*., 2023). In addition, the dysfunction of the pineal gland, which is involved in the production of endogenous DMT, has been implicated in some neuropsychiatric disorders, such as autism spectrum disorders and meditation has been linked to enhanced fMRI signal intensity of the pineal gland (Shomrat & Nesher, 2019; Barker, 2022; Plini *et al*., 2025). This raises the question, could, in some patient subgroups mindfulness- interventions be used as an alternative to exogenous hallucinogen microdosing?

In conclusion, there is not sufficient evidence to give recommendations on psychedelic use for ADHD. The evidence is inconclusive and scarce. In addition, it is not known whether patients, whose depressive symptoms have responded positively to ketamine, also have suffered from ADHD and to what extent. Also, no research was found on how psychedelics affect patient subgroups with possibly different etiopathological mechanisms behind their symptoms. As it has been proposed that ACEs can cause ADHD, and there is evidence that psychedelics might help PTSD patients, it might be important to study the effects of psychedelics in this patient group separately. Although only six studies fulfilled the inclusion criteria for this study, they bring out valuable implications for further research.
